# Supplementary material for: Brain-wide connectome inferences using functional connectivity MultiVariate Pattern Analyses (fc-MVPA)
Source: PLoS Comput Biol. 2022 Nov 15;18(11):e1010634. doi: 10.1371/journal.pcbi.1010634 (PMC9707802; doi:10.1371/journal.pcbi.1010634)
Supplement: S1 Appendix — (DOCX) [file pcbi.1010634.s001.docx]

**Appendix I. Efficient computation of multivariate patterns**

A diagram of the computational procedure followed to compute eigenpattern scores is shown in Fig S1.


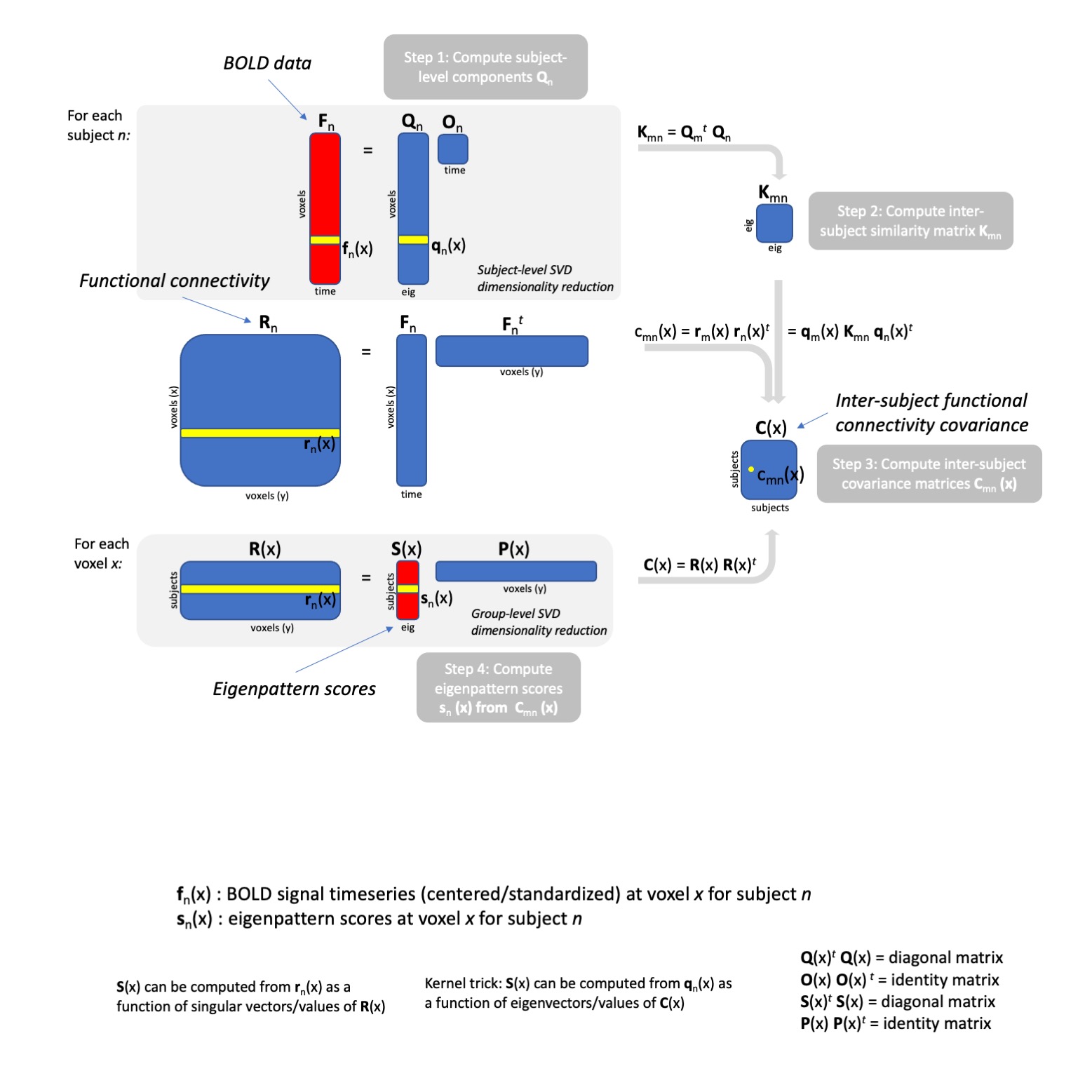


S1 Fig. Efficient computation of eigenpattern scores. Schematic summarizing the steps involved in computing eigenpattern scores for each subject. First, the procedure loops over each subject to decompose the voxels-by-time BOLD data matrix using Singular Value Decomposition (SVD) into orthogonal factors Q_n_. Them the intersubject similarity between these orthogonal factors is computed. Last, the procedure loops over each voxel to compute: a) the subject-by-subject functional connectivity covariance matrix C(x); and b) the associated eigenpattern scores by decomposing this covariance matrix using SVD.

Following the general voxel-to-voxel analysis procedure described by Whitfield-Gabrieli and Nieto-Castanon [18], we first characterize separately for each subject the voxel-to-voxel correlations $r_{n}\left( x,y \right)$ (one voxels-by-voxels matrix for each subject) without loss of generality or precision using a reduced set of orthogonal (not orthonormal) components $q_{n,i}\left( x \right)$, with one volume per subject and component, defined from a Singular Value Decomposition (SVD) of the preprocessed and normalized BOLD timeseries $\tilde{f}\left( x,t \right)$:

| $q_{n,m}(x) \vert r_{n}(x,y)\equiv\sum_{t=1}^{T} \tilde{f}_{n}\left( x,t \right)\cdot\tilde{f}_{n}\left( y,t \right)=\sum_{m=1}^{M} q_{n,m}\left( x \right)\cdot q_{n,m}\left( y \right)$ |  |
| --- | --- |

(note: *x* and *y* index over voxels, while *t, i*, and *n* index over scans, components, and subjects, respectively). The number of components *M* necessary to fully characterize (without any dimensionality reduction) the voxel-to-voxel correlation matrix is determined by the effective degrees of freedom of the original timeseries (approximately equal to the number of scans minus the number of regressed components multiplied by the proportion of the frequency spectrum covered by the band-pass filter preprocessing step), so the resulting components of the voxel-to-voxel covariance can always be stored using only a portion of the storage necessary to store the original BOLD timeseries.

We also compute from these components and store the cross-covariance matrix ***K***, characterizing the spatial similarity between the *m_1_*-th SVD component from subject *n_1_* and the *m_2_*-th component from subject *n_2_*:

| \| $K_{n_{1},n_{2},m_{1},m_{2}}\equiv\int q_{n_{1},m_{1}}\left( x \right)\cdot q_{n_{2},m_{2}}\left( x \right) dx$ \|  \| \| --- \| --- \| | (A1.1) |
| --- | --- | --- | --- |

as well as the spatial-covariance matrix ***L***, characterizing the degree of smoothness of these components:

| \| $L_{{i,j,n}_{1},n_{2}}=\sum_{m_{1},m_{2}=1}^{M} K_{n_{1},n_{2},m_{1},m_{2}}\cdot\int\frac{\partial}{\partial x_{i}}q_{n_{1},m_{1}}\left( x \right)\cdot\frac{\partial}{\partial x_{j}}q_{n_{2},m_{2}}\left( x \right) dx$ \|  \| \| --- \| --- \| | (A1.2) |
| --- | --- | --- | --- |

After computing $\boldsymbol{K}_{n_{1},n_{2}}$ and $q_{n,m}\left( x \right)$ for all subjects, we can then easily compute, from these measures alone and separately for each voxel, the voxel-specific subjects-by-subjects cross-covariance $C_{n_{1},n_{2}}\left( x \right)\equiv\int r_{n_{1}}\left( x,y \right)\cdot r_{n_{2}}\left( x,y \right) dy$, characterizing the between-subjects variability in functional connectivity maps between this voxel and the rest of the brain, as:

| \| $C_{\boldsymbol{n}_{\boldsymbol{1}}\boldsymbol{,}\boldsymbol{n}_{\boldsymbol{2}}}\left( x \right)\equiv\boldsymbol{r}_{n_{1}}\left( x \right)\cdot\boldsymbol{r}_{n_{2}}^{t}\left( x \right)=\sum_{m_{1},m_{2}=1}^{M} q_{n_{1},m_{1}}\left( x \right)\cdot q_{n_{2},m_{2}}\left( x \right)\cdot K_{n_{1},n_{2},m_{1},m_{2}}$ \|  \| \| --- \| --- \| | (A1.3) |
| --- | --- | --- | --- |

From the definition of ***C****(x)* above and Equation 5, it follows that:

| \| $\boldsymbol{C}\left( x \right)\boldsymbol{=R}\left( x \right)\boldsymbol{\cdot}{\boldsymbol{R}\left( x \right)}^{t}\boldsymbol{=S}\left( x \right)\cdot\boldsymbol{D}^{2}\left( x \right)\cdot\boldsymbol{S}^{t}\left( x \right)$ \|  \| \| --- \| --- \| | (A1.4) |
| --- | --- | --- | --- |

This means that we can directly compute ***S*** and ***D*** as the eigenvectors and squared root of eigenvalues, respectively, of the semidefinite positive matrix ***C****(x)*, and from this the eigenpattern scores:

| \| $\boldsymbol{s}_{n}\left( x \right)\boldsymbol{=}\boldsymbol{e}_{n}\cdot\boldsymbol{R}\left( x \right)\cdot\boldsymbol{P}\left( x \right)\cdot\boldsymbol{D}^{\boldsymbol{-1}}\left( x \right)=\boldsymbol{e}_{n}\cdot\boldsymbol{S}\left( x \right)$ \|  \| \| --- \| --- \| | (A1.5) |
| --- | --- | --- | --- |

where ***e****_n_* is a unit N-element vector with 1 in the n-th position and 0’s otherwise. Overall, this procedure means that we can compute the desired eigenpattern scores $\boldsymbol{s}_{n}\left( x \right)$ at a particular location *x* directly from an eigenvector decomposition of ***C****(x)* (Equation A1.4), which, in turn, can be computed only from $\boldsymbol{K}_{n_{1},n_{2}}$ and $q_{n,i}\left( x \right)$ (Equation A1.3), without ever having to undertake the considerably more time-consuming operations involved in explicitly computing or storing $\boldsymbol{R}\left( x \right)$ or $\boldsymbol{P}\left( x \right)$*.*

The computation of $\boldsymbol{C}\left( x \right)$ and $\boldsymbol{s}_{n}\left( x \right)$ is then iterated for each voxel *x* to create the full set of component volumes (one file per subject and component) storing the eigenpattern scores $\boldsymbol{s}_{n}\left( x \right)$. Note that the number of operations involved in these computations for any given voxel is independent of the number of voxels, and hence the total number of operations will scale only linearly with the total number of voxels in the analysis, as opposed to quadratically as in the original formulation, making the computation of the MVPA eigenpattern scores a considerably faster procedure.
