# Supplementary material for: Brain-wide connectome inferences using functional connectivity MultiVariate Pattern Analyses (fc-MVPA)
Source: PLoS Comput Biol. 2022 Nov 15;18(11):e1010634. doi: 10.1371/journal.pcbi.1010634 (PMC9707802; doi:10.1371/journal.pcbi.1010634)
Supplement: S2 Appendix — (DOCX) [file pcbi.1010634.s002.docx]

**Appendix II. Preprocessing and analysis of resting state functional data**

We performed fc-MVPA analyses to identify gender-related differences in functional connectivity using a publicly available dataset (Cambridge 1000-connectomes dataset, n=198; available at NITRC *fcon_1000*). All analyses are performed using CONN21 and SPM12. The functional data was realigned using SPM12 realign & unwarp procedure [1], where all scans are coregistered and resampled to a reference image (first scan of the first session) using b-spline interpolation. Temporal misalignment between different slices of the functional data was corrected using SPM12 slice-timing correction (STC) procedure [2]. Potential outlier scans were identified using ART [3] as acquisitions with framewise displacement above 0.5mm or global BOLD signal changes above 3 standard deviations [4]. Functional and anatomical data were normalized into standard MNI space and segmented into grey matter, white matter, and CSF tissue classes using SPM12 unified segmentation and normalization procedure [5]. Last, functional data was smoothed using spatial convolution with a Gaussian kernel of 8mm full width half maximum (FWHM). In addition, default CONN denoising steps were performed including the regression of session effects and their first order derivatives (2 factors), motion parameters and their first order derivatives (12 factors), outlier scans (below 30 components), white matter (5 components), and CSF timeseries (5 components) as potential confounding effects, detrending, and band-pass filtering between 0.008Hz and 0.09Hz [6-9]. After outlier removal there were an average of 114.6 functional scans/acquisitions per subject (ranging between 89 to 119), and after denoising and band-pass filtering the residual effective degrees of freedom averaged 43.6 (ranging between 31.0 to 45.8). Residual inter-scan motion (also computed after outlier removal) was 0.087mm on average, with 0.012 mm higher motion (T(196) = 2.75, p = 0.006565) in male subjects (N=75) compared to female subjects (N=123).

Quality control plots were created using CONN based on the analysis of functional connectivity values (Pearson correlation coefficients between the BOLD before and after denoising at each pair of nodes) in a graph formed by 1,000 nodes randomly selected within gray matter voxels. Functional connectivity (FC) distributions indicated reasonably centered distribution of voxel-to-voxel correlation values after denoising (see Fig S2 bottom plot), with global correlation, the average of all correlations between pairs of voxels separately for each subject, equal to GCOR = 0.029 ±0.012 (average ± standard deviation across subjects), which were as expected considerably lower and more stable across subjects compared to global correlations before denoising GCOR = 0.467 ±0.099. Additional QC-FC quality control analyses (shown in Fig S3) computed the estimated strength of residual intersubject correlations between measures of subject motion (average framewise displacement) and functional connectivity strength at each edge within the same random graphs [10-11]. These plots also indicate appropriate levels of denoising, with distributions of motion-connectivity correlations after denoising (gray area in Fig S3 bottom plot) similar -95.6% match- to those expected by chance (red dashed lines). Similarly, QC-FC associations between functional connectivity strength and the number of valid scans remaining for each subject after outlier detection showed negligible effects after denoising, with 97.2% match with the null hypothesis distribution.

First-level analyses included fc-MVPA, with subject-level dimensionality reduction set to 64 dimensions, and the number of eigenpatterns estimated at each voxel set to 100. Second-level analyses then used the first 10 components among the 100 estimated eigenpatterns in order to maintain a conservative 20:1 ratio of subjects-to-components. The resulting eigenpattern score volumes were entered into a multivariate second-level General Linear Model analysis with 198 samples (subjects) and 10 observations (eigenpattern scores). The model included two factors: gender (categorical factor with two levels) and subject motion (a continuous factor, encoding residual average framewise displacement after removal of outlier scans for each subject). The contrast [-1 1 0] testing gender-related differences in connectivity controlling for subject motion was evaluated using F(10,186) statistics. Threshold Free Cluster Enhancement scores [12] were estimated using CONN’s default values for F- statistics H=1, E=0.5, and H_min_=1. Non-parametric randomization test [13] with 1,000 simulations were used to determine TFCE score statistics under the null hypotheses. Analysis results were thresholded at a family-wise error corrected p-FWE < 0.05 level. Last, for effect-size estimation we computed $\boldsymbol{h}_{\mathrm{map}}$ using Equation 9 for each significant cluster, characterizing the difference in connectivity between male and female subjects when compared at the same level of the control covariate (subject motion).

| 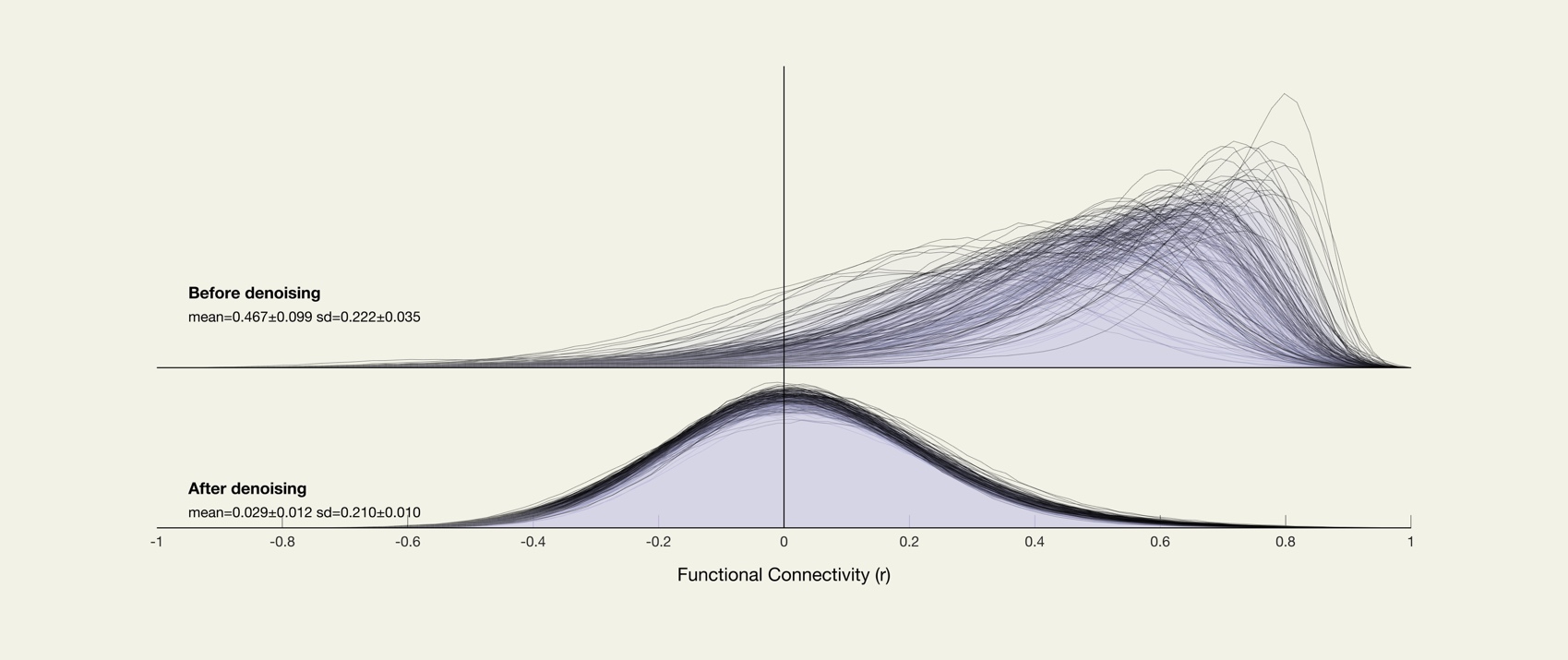 |
| --- |

S2 Fig. Quality control plots of functional connectivity (FC) histograms. Distributions of Pearson correlation values between the BOLD signal at 1,000 random nodes within gray matter voxels, computed separately for each subject before denoising (top plots) and after denoising (bottom plots). Distributions after denoising appeared well centered (distributions mode close to r=0 values) and homogeneous (distributions shape similar across subjects), both markers indicative of appropriate levels of denoising of the BOLD signal.

| 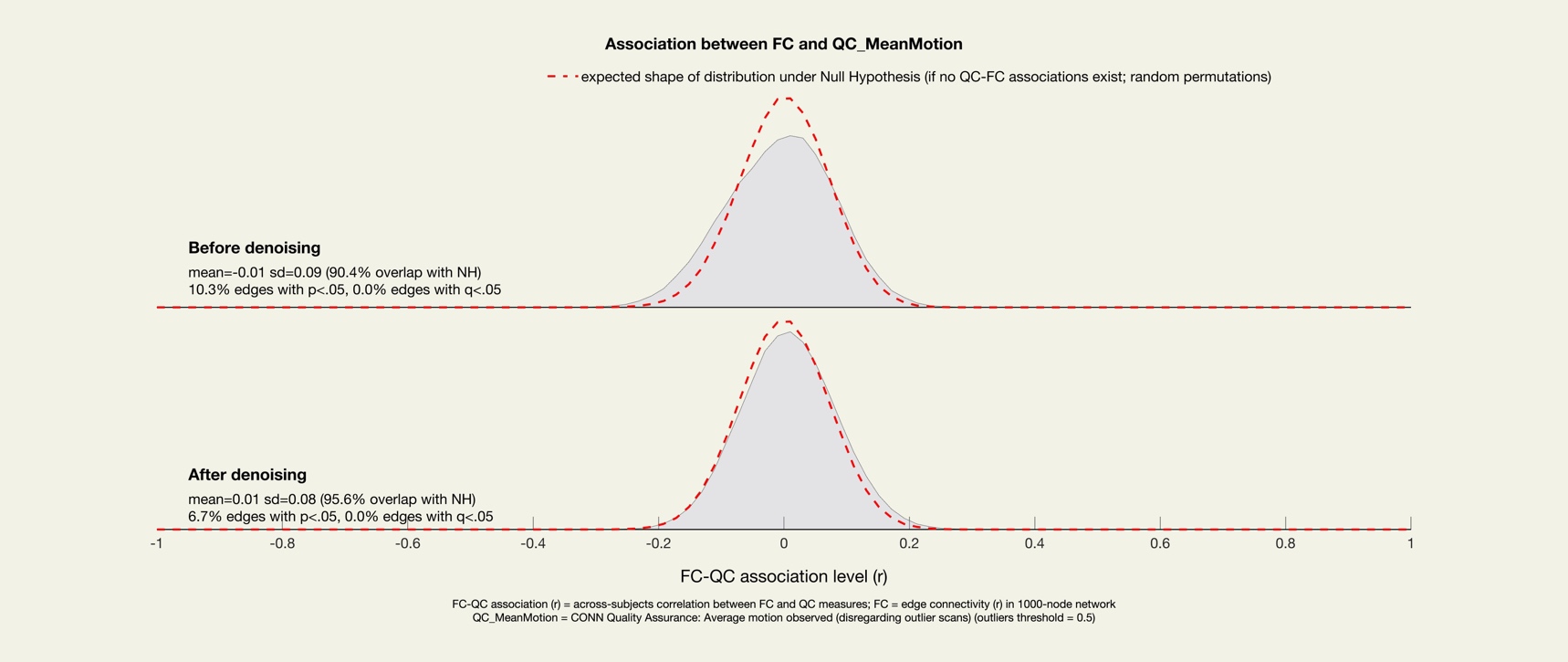 |
| --- |

S3 Fig. Quality control plots of QC-FC correlations. Distributions of Pearson intersubject correlation values between the functional connectivity strength at all edges in a 1000-node graph and a measure of subject motion (average framewise displacement), computed separately before denoising (top plots) and after denoising (bottom plots). Red dashed lines show the expected distribution of QC-FC correlations in the absence of meaningful associations between connectivity strength and subject motion (obtained using permutation analyses of the same data). QC-FC distributions show a good match with the expected distribution under the null hypothesis (95.6% overlap between the two distributions) after denoising, with percent match above 95% indicative of appropriate levels of denoising of the BOLD signal [11].

**References**

1. Andersson JLR, Hutton C, Ashburner J, Turner R, Friston K. Modelling geometric deformations in EPI time series. NeuroImage. 2001; 13(5):903-19.
2. Henson RNA, Buechel C, Josephs O, Friston KJ. The slice-timing problem in event-related fMRI. NeuroImage. 1999;9:125
3. Whitfield-Gabrieli S, Nieto-Castanon A, Ghosh, SS. Artifact detection tools (ART). Cambridge, MA. Release Version. 2011;7(19):11.
4. Power JD, Mitra A, Laumann TO, Snyder AZ, Schlaggar BL, Petersen SE. Methods to detect, characterize, and remove motion artifact in resting state fMRI. Neuroimage. 2014;84:320-41.
5. Ashburner J, Friston KJ. Unified segmentation. NeuroImage. 2005;26:839–51.
6. Friston KJ, Williams S, Howard R, Frackowiak RS, Turner R. Movement‐related effects in fMRI time‐series. Magnetic resonance in medicine. 1996;35(3):346-55.
7. Behzadi Y, Restom K, Liau J, Liu TT. A component based noise correction method (CompCor) for BOLD and perfusion based fMRI. Neuroimage. 2007;37(1):90-101.
8. Chai XJ, Nieto-Castañón A, Öngür D, Whitfield-Gabrieli S. Anticorrelations in resting state networks without global signal regression. Neuroimage. 2012;59(2):1420-28.
9. Nieto-Castanon A. FMRI denoising pipeline. In: Handbook of functional connectivity Magnetic Resonance Imaging methods in CONN. Hilbert Press; 2020. pp 17-25.
10. Ciric R, Wolf, DH, Power JD, Roalf DR, Baum GL, Ruparel K, et al. Benchmarking of participant-level confound regression strategies for the control of motion artifact in studies of functional connectivity. Neuroimage. 2017;154:174-87.
11. Nieto-Castanon, A. (2022). Preparing fMRI Data for Statistical Analysis. arXiv preprint arXiv:2210.13564.
12. Smith SM, Nichols TE. Threshold-free cluster enhancement: addressing problems of smoothing, threshold dependence and localisation in cluster inference. Neuroimage. 2009;44(1):83-98.
13. Bullmore ET, Suckling J, Overmeyer S, Rabe-Hesketh S, Taylor E, Brammer MJ. Global, voxel, and cluster tests, by theory and permutation, for a difference between two groups of structural MR images of the brain. IEEE transactions on medical imaging. 1999;18(1):32-42.
