# Supplementary material for: Brain-wide connectome inferences using functional connectivity MultiVariate Pattern Analyses (fc-MVPA)
Source: PLoS Comput Biol. 2022 Nov 15;18(11):e1010634. doi: 10.1371/journal.pcbi.1010634 (PMC9707802; doi:10.1371/journal.pcbi.1010634)
Supplement: S1 Table — (DOCX) [file pcbi.1010634.s003.docx]

**Table S1. Glossary of terms in manuscript equations**

| **Term** | **Dimensionality** | **Description** |
| --- | --- | --- |
|  |  |  |
| ***Constants/indexes*** | | |
| *M* | Scalar | Number of voxels |
| *N* | Scalar | Number of subjects |
| *K* | Scalar | Number of eigenpatterns in fc-MVPA |
| *L* | Scalar | Number of predictor variables in GLM |
| *J* | Scalar | Number of individual contrasts in GLM |
| *n* | Scalar | Index to individual subject |
| *k* | Scalar | Index to individual eigenpattern |
| $x,y$ | Scalars | Index to individual voxels |
| $\Omega$ | Set | Set of all voxels within an individual cluster |
|  |  |  |
| ***Measures of connectivity*** | | |
| $\boldsymbol{R}\left( x \right)$ | Matrix [NxM] | Functional connectivity with voxel *x* |
| $\boldsymbol{r}_{n}\left( x \right)$ | Vector [1xM] | Functional connectivity with voxel *x* for subject *n* (a row of $\boldsymbol{R}\left( x \right)$) |
| $r_{n}\left( x,y \right)$ | Scalar | Functional connectivity between voxels *x* and *y* for subject *n* |
|  |  |  |
| ***Eigenpattern & Eigenpattern scores*** | | |
| $\boldsymbol{S}\left( x \right)$ | Matrix [NxK] | Eigenpattern scores |
| $\boldsymbol{s}_{n}\left( x \right)$ | Vector [1xK] | Eigenpattern scores (individual row of $\boldsymbol{S}\left( x \right))$ |
| $\boldsymbol{P}\left( x \right)$ | Matrix [MxK] | Between-voxels contrast matrix in fc-MVA  Eigenpatterns matrix in fc-MVPA |
| $\boldsymbol{D}\left( x \right)$ | Matrix [KxK] | Diagonal matrix of singular values |
|  |  |  |
| ***General Linear Model*** | | |
| $\boldsymbol{G}$ | Matrix [NxL] | Design matrix |
| $\boldsymbol{g}_{n}$ | Vector [1xL] | Vector of predictor variables for subject *n* (individual row in ***G***) |
| $\varepsilon_{n}\left( x,y \right)$ | Scalar | Error term for each subject in fc-MUA GLM |
| $\boldsymbol{\varepsilon}_{n}\left( x \right)$ | Vector [1xM] | Error term in fc-MVA GLM |
| ${\tilde{\boldsymbol{\varepsilon}}}_{n}\left( x \right)$ | Vector [1xK] | Error term in fc-MVPA GLM |
| $\sigma\left( x,y \right)$ | Scalar | Standard deviation of error term in fc-MUA GLM |
| $\boldsymbol{\Sigma}\left( x \right)$ | Matrix [MxM] | Cholesky factor of error covariance in fc-MVA GLM |
| $\tilde{\boldsymbol{\Sigma}}\left( x \right)$ | Matrix [KxK] | Cholesky factor of error covariance in fc-MVPA GLM |
| $\boldsymbol{b}\left( x,y \right)$ | Vector [Lx1] | Estimated regressor coefficients in fc-MUA GLM |
| $\boldsymbol{B}\left( x \right)$ | Matrix [LxM] | Estimated regressor coefficients in fc-MVA GLM |
| $\tilde{\boldsymbol{B}}\left( x \right)$ | Matrix [LxK] | Estimated regressor coefficients in fc-MVPA GLM |
| $\boldsymbol{C}$ | Matrix [JxL] | Between-subjects contrast matrix |
| $\boldsymbol{c}_{j}$ | Vector [1xL] | Between-subjects contrast vector (individual row of ***C***) |
| $F(x)$ | Scalar | Parametric F statistic |
| $\lambda$ | Scalar | Parametric Wilks’ Lambda statistic |
| a | Scalar | Number of eigenpatterns in fc-MVPA GLM |
| b | Scalar | Error degrees of freedom |
| c | Scalar | Hypothesis degrees of freedom |
| $\boldsymbol{W(}x\boldsymbol{)}$ | Matrix [KxK] | Error sum of squares |
| $\boldsymbol{H(}x\boldsymbol{)}$ | Matrix [KxK] | Hypothesis sum of squares |
| $\boldsymbol{h}_{\mathrm{eig}}\left( \Omega\right)$ | Vector [1xK] | Contrast effect-size per eigenpattern |
| $\boldsymbol{h}_{\mathrm{scores}}\left( \Omega\right)$ | Vector [1xN] | Contrast subject scores |
| $\boldsymbol{h}_{\mathrm{map}}\left( \Omega\right)$ | Vector [1xM] | Contrast effect-size per voxel |
