# Supplementary material for: Brain-wide connectome inferences using functional connectivity MultiVariate Pattern Analyses (fc-MVPA)
Source: PLoS Comput Biol. 2022 Nov 15;18(11):e1010634. doi: 10.1371/journal.pcbi.1010634 (PMC9707802; doi:10.1371/journal.pcbi.1010634)
Supplement: Attachment — Submitted filename: ReviewersResponse.pdf [file pcbi.1010634.s004.pdf]

***Reviewer #1**: The author has previously provided a new tool for performing MVPA for inference in the context of functional connectivity (fc-MVPA). Here, the author describes fc-MVPA in detail, including its formal definition and properties, an empirical example to demonstrate its use and interpretation, and a simulation to demonstrate its valid control of false positive rates and its sensitivity under various conditions. Overall, this work provides a valuable and comprehensive overview for understanding an important tool for functional connectivity analysis. The description of how to interpret various components (e.g., effect size reporting, eigenpatterns) is particularly valuable.*

I only had a few minor suggestions.

"Classical MVPA analyses (e.g. Norman et al. 2006) attempt to predict, from these or other searchlight patterns, properties of the subjects (e.g. patients vs. controls) or properties of the experimental paradigm (e.g. pre- vs. after- intervention). These are often referred to as backward models (Haufe et al. 2014), and typically focus on machine learning classification models. Unlike those forms of MVPA, fc-MVPA uses instead a forward model of the data, attempting to predict the shape of these searchlight patterns from subject and experimental paradigm information." (p 7)

The use of "predict" here for both predictive and explanatory modeling could be a little confusing. Although "predict" is historically used for explanatory procedures (e.g., regression), they do not actually typically involve any prediction (e.g., of unseen data) and it is now becoming more accepted to reserve "predict" for machine learning contexts. Consider something like "estimate the relationship between these searchlight patterns" instead.

I would like to thank the reviewer for this excellent observation, I have now changed the text to avoid the use of the term "prediction" in the context of regression models and use instead the more common terms "independent/explanatory" variables and "dependent/outcome" variables instead. The text now reads the following:

"…Most MVPA methods focus on the relationship between properties of these multivariate patterns, characterizing a subject's mental state, and either static (e.g. patients vs. controls) or dynamic properties of the experimental design (e.g. pre- vs. after- intervention, task vs. rest, etc.), while varying in how exactly these relationships are modeled or analyzed. Classical MVPA analyses (e.g. Norman et al. 2006, Pereira et al. 2009) attempt to estimate, from these or other searchlight patterns, properties of the experimental paradigm. These MVPA analyses are often referred to as backward models (Haufe et al. 2014), and typically use machine learning classification models embedded in a cross-validation framework to decode information about a subject's mental state from their activation patterns. In this popular class of MVPA models searchlight patterns act as independent/explanatory variables, while known subject or experimental properties act as dependent/outcome variables. Unlike those forms of MVPA, fc-MVPA uses instead a forward model of the data, attempting to estimate the shape of these

searchlight patterns from known subject or experimental properties (switching the role of dependent/independent variables)…"

Can the author provide a reference to any similar formal descriptions available for traditional "backwards model" MVPA in the literature? I found myself wanting to consult an additional text to see whether there were analogues for some details of the presented procedure (e.g., choice of a spatial basis).

Some great papers describing a traditional "backwards model" MVPA are perhaps Norman et al. 2006, and Pereira et al. 2009. I have included these references in the text in page 3

Recent work has shown that multivariate inferential methods have better power than (mass) univariate methods for functional connectivity. If the author wants, it may be helpful for empirically motivating the present work: Noble, S., Mejia, M., Zalesky, A., & Scheinost, D. (2021). Leveling up: improving power in fMRI by moving beyond cluster-level inference. BioRxiv.

Thank you for this reference. I have now included this in the text (now Noble et al. 2022) when discussing Multivariate cNBS in page 2 and when discussing multivariate inferences in general in page 3

***Reviewer #2**: This manuscript presents foundational materials and empirical results to support the use of MVPA with BOLD low-frequency MRI data to allow for voxel-wise comparisons of functional connectivity differences using the standard general linear model (e.g., fc-MVPA). The code and software implementation of the proposed MVPA technique has been made widely available by the submitting author, and the technique, as implemented in the MATLAB CONN toolbox (see https://www.nitrc.org/projects/conn), has been used in no less than 26 peer-reviewed publications with moderate to high impact factor ratings.*

This manuscript provides valuable clarity into the theoretical underpinnings involved in generating eigenpatterns from singular value decomposition procedures, which further reinforces the utility of the fc-MVPA technique. Additionally, the manuscript provides critical support for the technique through simulation analyses to determine the impact of eigenpattern selection on the validity and sensitivity of the technique with various sample sizes.

Overall, I found the study to be interesting and the technique to be a valuable addition to the neuroimaging community, as it potentially addresses multiple problems inherent to traditional parcellation-based neuroimaging techniques. I found very little to fault in the manuscript aside from a few grammatical mistakes, though I would have liked to have seen more discussion on how regression models using fc-MVPA might be cross-validated using the existing CONN toolbox or code.

I would like to thank the reviewer for these generous comments and great suggestion. I totally agree that the manuscript would benefit from at least some discussion on the uses of cross-validation in the context of fc-MVPA, so, while this is a complex topic, I have now added at least a brief discussion on the use of cross-validation for unbiased effect-size estimation in page 13, for parameter optimization in page 11, as well as some details about extending the computation of eigenpattern scores to other "out-of-sample" subjects on page 16. I have also submitted the manuscript through AJE editor services to identify and correct hopefully most remaining grammatical errors.

It may be beyond the scope of the manuscript to provide more end-user friendly information on the implementation of the fc-MVPA technique, but the authors are encouraged to provide this information as either supplementary materials or link to materials in the toolbox's NITRC database listing.

I would also like to thank the reviewer for this suggestion. We have updated CONN´s manual (available at https://www.conn-toolbox.org/resources/documentation) to add more details to the description of the steps involved in running a fc-MVPA analysis in CONN, and also updated the development version of CONN in GitHub (https://www.github.com/alfnie/conn) to match the nomenclature and definitions used in this manuscript. Both of these will be packaged as well into the next public release of CONN in NITRC (https://www.nitrc.org/projects/conn) in the next few months after

we´ve had some time to receive feedback from users of the development version and address potential usability issues, bugs, or any other related comments/suggestions.

***Reviewer #3**: Generally, I find that having an article detailing this work is a useful endevour and that a report on this is missing in the literature.*

At the moment, the article is between a tutorial targetting researchers who will apply the method and a mathematical background information on the method developed targetting a more methodological population.

It is regrettable that the documentation of the software package is not more deeply integrated to the description of the method, for instance with a Jupyter book/notebook.

I would like to thank the reviewer for these comments and for this great suggestion. I entirely agree that a Jupiter notebook jointly describing the method and software would be very useful. We have already started creating an executable notebook describing fc-MVPA using Matlab´s Live Scripts, and this will be made available at the www.conn-toolbox.org/tutorials section over the next few months (ideally, I would like to follow this up by adding similar notebooks for other methods implemented in CONN as I agree this would be a great addition to the learning resources available to CONN users). In addition, and as mentioned in Reviewer #2 response and following CONN's standard procedures for documentation, we have also updated the software manual (available at https://www.conn-toolbox.org/resources/documentation) to add more details to the description of the steps involved in running a fc-MVPA analysis in CONN, and also updated the development version of CONN in GitHub (https://www.github.com/alfnie/conn) to match the nomenclature and definitions used in this manuscript. Both of these will be packaged as well into the next public release of CONN in NITRC (https://www.nitrc.org/projects/conn) in the next few months after we´ve had some time to receive feedback from users of the development version and address potential usability issues, bugs, or any other related comments/suggestions.

I also found confusing the name of the method, as MVPA is a well established method in brain imaging analysis but does not rely on the same principles as fc-MVPA. This is bound to induce confusion in the minds and in the literature. I recommend that the method is renamed avoiding the term "pattern" for instance, or using MCOR as a core acronym.

While it is true that one particular form of MVPA (backwards classification models using cross-validated predictions) has become widely popular, the general class of MVPA methods is much larger (see Haxby, 2012 for a delightful discussion of the origins of MVPA). In particular, MVPA encompasses a variety of methods that focus on analyzing neural responses in terms of multivariate patterns of activation. In this context, fc-MVPA simply switches the focus of traditional MVPA analyses to consider at each voxel, instead of the pattern of activation around it, the pattern of connectivity between this voxel and the rest of the brain. In addition, fc-MVPA departs from most of the common MVPA applications in that it uses a "forward model" of the data (where known subject

and experimental features are used as explanatory/independent variables, while the subject's connectivity patterns are used as outcome/dependent variables), while many MVPA applications use a "backward model" of the data (where the role of dependent/independent variables is reversed). I imagine that, in the future, other forms of fc-MVPA analyses may be suggested departing from the specifics of the method proposed in this paper (e.g. they may choose to use backwards classification models popular in the context of functional activation analyses instead of the forward models proposed here for brain-wide inferences). To clarify this point I have extended the discussion in page 3 regarding the different forms of MVPA methods, and how fc-MVPA fits within them. The modified paragraph now reads the following:

"This paper proposes functional connectivity Multivariate Pattern Analysis (fc-MVPA), an alternative approach to the analysis of the brain-wide connectome at the resolution of individual voxels, that overcomes the difficulties of brain-wide connectome analyses using multivariate pattern analysis techniques. Like other MVPA approaches in neuroimaging, fc-MVPA follows a general searchlight procedure (e.g. Kriegeskorte et al. 2006), but instead of focusing on the pattern of activation surrounding each voxel, fc-MVPA considers separately for each voxel the entire multivariate pattern of functional connections between this voxel and the rest of the brain. Most MVPA methods focus on the relationship between properties of these multivariate patterns, characterizing a subject's mental state, and either static (e.g. patients vs. controls) or dynamic properties of the experimental design (e.g. pre- vs. after- intervention, task vs. rest, etc.), while varying in how exactly these relationships are modeled or analyzed. Classical MVPA analyses (e.g. Norman et al. 2006, Pereira et al. 2009) attempt to estimate, from these or other searchlight patterns, properties of the experimental paradigm. In this popular class of MVPA models searchlight patterns act as independent/explanatory variables, while known subject or experimental properties act as dependent/outcome variables. These MVPA analyses are often referred to as ***backward models*** *(Haufe et al. 2014), and typically use machine learning classification models embedded in a cross-validation framework to decode information about a subject's mental state from their activation patterns. Unlike those forms of MVPA, fc-MVPA uses instead a* ***forward model*** *of the data, attempting to estimate the shape of these searchlight patterns from known subject or experimental properties (switching the role of dependent/independent variables). In addition to being advantageous in terms of the interpretability of model weights, forward models also enable us to frame brain-wide connectome inferences in the context of the General Linear Model (GLM), one of the most widely used inferential statistical methods in neuroimaging, and use powerful multivariate inferences (Noble et al. 2022) to directly address researcher's hypotheses. Specifically, this approach allows us to make statistical inferences about individual voxels in the brain regarding the shape of their functional connectivity patterns (e.g. is the shape of the functional connectivity pattern between a voxel and the rest of the brain different in patient vs. control subjects?), and then repeat these analyses across all voxels within the brain extending these inferences to the entire connectome (e.g. is the shape of functional connectivity patterns different in patient vs. control subjects anywhere in the brain?)."*

I have also added a new paragraph in page 23 in the conclusions section emphasizing/summarizing the link between fc-MVPA and other MVPA approaches. This paragraph reads the following:

“Fc-MVPA extends or complements other MVPA approaches commonly used in neuroimaging in three different ways: first, to characterize a subject’s mental state, instead of the patterns of activation surrounding each voxel considered by many MVPA applications, fc-MVPA considers the patterns of connectivity between each voxel and the rest of the brain; second, instead of a backward model focusing on decoding known properties of a subject or of the experimental paradigm, fc-MVPA uses a forward model focusing on testing researcher’s hypothesis about the subject’s connectivity state across the entire connectome (brain-wide connectome inferences); and third, in addition to the above inferential framework, fc-MVPA also provides a model-free characterization of the sources of intersubject heterogeneity in connectivity patterns.”

For the reasons above, while I understand and share the reviewer’s concern, I prefer to maintain the current fc-MVPA name as I believe it is important to highlight the strong associations between fc-MVPA and other MVPA methods, as well as to maintain continuity across publications which have already used or referred to this method as MVPA.

The simulation section could be pushed to an appendix. I found the numbers chosen fairly extreme (eg FWHM either 0 or 100). Choices that span a more realistic fMRI analysis setting would be preferable.

That is a very good observation as well. I have added new simulations to cover the range of FWHM values from 0 to 25 voxels in order to more closely represent reasonable/realistic values. Those simulations have been added to the results described in the text and shown in Figures 7 and 8. I tentatively decided against moving this section to the appendix because I feel the method’s validation is one of the core contributions of the paper, but I am perfectly fine reconsidering this if the reviewer feels strongly about it.

Last, I think that leaving the method without a reasonable way to estimate the number of eigen components to select is very risky. Researchers will try several values and most likely only report the "most interesting" results - leading to some form of p-hacking. There are a number of possible methods to at least guide this choice, at least the danger of selecting a value after having seen the statistical results should be described and avoided.

That is also an excellent observation. I agree with the reviewer’s concern that, lacking a reasonable way to choose the number of eigenpatterns, some researchers may be tempted to “p-hack” their way to statistical significance. I have included an explicit discussion of this issue in page 11, and I have also included an explicit recommendation for the number of eigenpatterns, some thoughts on when it may be justifiable to depart from these recommended values, as well as a brief description of multiple comparison correction approaches when evaluating the results across multiple choices of this parameter. In general I recommend exploration and proper reporting of the results as a

better way to discourage p-hacking than restricting user-choices in processing or analysis parameters, so I have tried to express that general belief through my recommendations. This section now reads the following:

"Another important choice that remains when using fc-MVPA in the context of brain-wide connectome inferences is to select k, the dimensionality of s_n (x) or the number of eigenpattern scores used to represent functional connectivity at each voxel. As it will be discussed in more detail in the Simulations section below, there is no "correct" choice of this parameter, and fc-MVPA inferences remain valid for all possible values of this parameter. Nevertheless it is important that this choice is made a priori and justified (e.g. from prior literature), and, if different values are tested/evaluated, the results of all these different evaluations should be reported (rather than reporting only the value that produces the best results for a particular analysis, which would inflate the chance of false positives). Regarding sensitivity of fc-MVPA inferences, choosing a low number of eigenpatterns value of k can be expected to improve sensitivity to detect relatively large or widespread effects of interest such as inter-network connectivity differences, while choosing a higher number of dimensions value of k can improve our ability to detect relatively smaller or marginal effects such as connectivity with smaller areas or subnetworks. In the absence of assumptions about the extent of the expected effects, A a reasonable balance is to scale the number of dimensions the choice of k with the dataset size (e.g. suggested 5:1, 10:1, or 20:1 ratios between N:k, the number of subjects in the analysis and the number of eigenpattern scores retained, Vittinghoff et al. 2006, Concato et al. 1995, Harrell et al. 1996), in order to maintain a reasonable sensitivity to identify large effects in small samples, and comparatively finer details in the analysis of larger samples. As with any other preprocessing or analysis choices, as long as the choice of k is made a priori, statistical inferences will remain valid. If, on the other hand, the value of k is selected a posteriori as the one that produced the "best" results among several possible values evaluated, an appropriate multiple-comparison correction should be used for statistical inferences (e.g. using a Bonferroni corrected cluster-level threshold p-FWE<0.05/10 if the results were selected among 10 different choices of k values, or using split or cross-validation procedures such as choosing k as the value that produces the "best" results in one half of the subjects and then basing statistical inferences on the analysis of the other half using the selected k value). For new analyses, and in the absence of any other rationale (e.g. based on prior literature, N:k ratios, or expected extent of effects) we recommend using a value of k=10, as that seems to suffice to cover a large proportion of the inter-subject variance in functional connectivity profiles (e.g. see Figure 4 results below). In all cases we encourage researchers to evaluate and report the robustness of their results to different choices of this parameter (e.g. as exploratory post-hoc analyses, without a need for additional multiple comparison corrections), as that will help other researchers build upon those results in future analyses and the field converge toward useful conventions"

Details:

Can the author better explain / comment on the following ?

** Eq. 1: \epsilon(x,y) seems to be implementing heterocedasticity across subjects, is OLS still the best minimal variance estimator ?*

Epsilon is the error/random term sampled from a zero-mean unit-variance Gaussian distribution, with sigma characterizing the (common) variance across subjects. The variance is assumed to be non-stationary (It can vary across seed and target voxels) but it is still constant across subjects (homoscedasticity, i.e. sigma does not depend on *n*), so OLS is still in this context the minimal variance / BLUE estimator, either from a univariate perspective as in Eq. 1 or from a multivariate perspective as in Eq. 2. I have now added a small clarification in page 5 regarding this point

** The index n seems to be indexing subjects in eq 1, but then in eq 2 the lenght of r_n(x,y) is the number of voxel y. It would be easier to just define the dimensions of these vectors and matrix. It seems that \bold{r_n(x)} is of dimension 1,p where p is the number of pixels, but it would be clearer with explicit dimensions*

** there are inconsistencies in what is a column and a row vector,*

** some matrix definitions come several lines after their use, it is generally ok but in this case these definitions come fairly late*

I would like to thank the reviewer for this recommendation. I have now added in Appendix III a glossary table of all terms included in the manuscript equations, indicating the dimensionality and interpretation of all vectors/matrices in the manuscript's equations. I have also made sure in all equations that the different terms are either already defined previously or explicitly defined shortly in the paragraph below each equation.

** Eq 5 would be easier to read as variances or squared norms of the corresponding matrices. Eg define the variance of the estimated effects and use this in Eq 5.*

I would like to thank the reviewer for this suggestion. Equation 5 have now been simplified to more clearly reflect the definition of ***P***(x) from a SVD factorization of the matrix ***R****(x)*.

** Eq 8 doesnt seem to include the dimentionality reduction - ie the definition of H and W have not been updated - I may have missed something here.*

Matrices ***H****(x)* and ***W****(x)* are both functions of ***P***(x), which characterizes the choice of dimensionality reduction, so their definition remains the same both in the the context of fc-MVA and fc-MVPA analyses. I have clarified this point in the text now in page 9 and in the Appendix glossary table, where I specify which GLM terms are specific to fc-MUA, fc-MVA, or fc-MVPA analyses (and which are common across all three cases).

** Ω is not precisely defined ("each significant cluster Ω") - it is unclear why we have to take integrals rather than sum which would make the material more accessible*

I would like to thank the reviewer for this suggestion. I have changed all integrations to summations, and I have added a more explicit definition of Omega in the text in page 11 and in the Appendix

** Between subjects contrasts are not necessarily one dimensional - eg think of the F statistics for difference between 3 groups, where the relevant contrast would be an "F-contrast" (eg \citep{Poline2007})*

The between-subjects contrast in this manuscript equations is already assumed to be a matrix (named ***C*** in these equations), while the vector ***c*** refers to the individual rows of the ***C*** matrix and it is used for effect-size estimation in order to quantify the individual components of the effect of interest in potentially-multivariate tests like an "F-contrast". I have now clarified the difference between the matrix ***C*** and the individual vectors $\boldsymbol{c}_j$ in the text and in the Appendix glossary table

** Effect sizes are difficult to understand for a non methodological oriented reader. If the article is aiming at the researchers who will apply the method, the should be better explained. More importantly, since all data are used to the SVD dimension reduction, they will be inflated. An estimation of this inflation should at least be provided (eg by separating the dataset into train and test sets), ideally a way to estimate non biaised effect sizes or correct for the bias would be provided.*

** I would try to relate the effect sizes to more interpretable values, for instance the correlation differences averaged across voxels, which would be a location dependent measure.*

I would like to thank the reviewer for this suggestion. I have now added a more thorough explanation of the interpretation of effect-sizes in pages 11&12, and discussed the potential use of cross-validation approaches in the context of fc-MVPA effect-size estimation in page 12.

** the author should be careful with the choice of colormap. It is well documented why jet / similar colormap should be avoided.*

I would like to thank the reviewer for this suggestion. I have changed the *jet* colormap in Figure 3 and used a *hot* colormap instead (other figures already use either a *parula* colormap or simpler two-color gradients in order to avoid the issues associated with *rainbow* colormaps such as *jet*).

** Fig 8: it would be easier to read to figure with a selection of say 3 or 4 number of components and show the ROC curves in 2D. At the moment, there are redundancies and also some of the curves are hard to see.*

I would like to thank the reviewer for this suggestion. I have modified both figures 7 & 8 to make the surfaces transparent, change the view angle closer to a 2D view, and fix the number of ROC curves shown, in order to make these results simpler to see and interpret.
